# Radiation Therapy Improves Survival in Patients with Distant Metastatic Head and Neck Squamous Cell Carcinoma: A Retrospective Study

**DOI:** 10.7150/jca.105254

**Published:** 2025-01-01

**Authors:** Mengwen Wang, Baitian Zhao, Amin Huang, Ming Song, Jia Wang, Panpan Liu

**Affiliations:** 1 State Key Laboratory of Oncology in South China, Guangdong Provincial Clinical Research Center for Cancer, Collaborative Innovation Center for Cancer Medicine, Sun Yat-sen University Cancer Center, Guangzhou 510060, China.; 2 Department of Medical Oncology, Sun Yat-sen University Cancer Center, Guangzhou 510060, China.; 3 Department of Head and Neck Surgery, Sun Yat-sen University Cancer Center, Guangzhou, Guangdong, China.; 4 Department of Clinical Trials Center, China.; 5 Department of medical oncology, the First Affiliated Hospital, Sun Yat-sen University, China.; 6 The Tenth Affiliated Hospital of Southern Medical University, Dongguan, Guangdong, 523059, China.

**Keywords:** distant metastasis, squamous cell carcinoma of the head and neck, survival, SEER program, radiation therapy

## Abstract

Background: The role of radiation therapy in patients with distant metastatic squamous cell carcinoma of the head and neck (mHNSCC) is unclear. In this study, we compare the differences in survival among mHNSCC patients treated with chemotherapy plus radiotherapy (RT) vs. chemotherapy alone.

Materials and Methods: This study included patients with distant mHNSCC recruited from 2 cohorts: The Surveillance, Epidemiology, and End Results (SEER [*N*=885]) database and a Chinese single-institution registry in Sun Yat-sen University Cancer Center (SYSUCC [*N*=60]). The SEER cohort included 600 patients received RT plus chemotherapy and 285 patients received chemotherapy alone; in the SYSUCC cohort, 40 patients received RT plus chemotherapy and 20 patients received chemotherapy alone were recruited. The period of data collection for the SEER study was from January 2010 to December 2015, and that for SYSUCC was from January 2010 to December 2020. The study's primary outcome was overall survival (OS), with disease-specific survival (DSS) as a secondary outcome.

Results: Of the 885 patients in the SEER cohort, the addition of RT to chemotherapy increased one-year OS from 46.6% to 56.0% compared with chemotherapy alone (*P* =0.009) and from 10.4% to 25.1% for three-year OS (*P* <0.001). Patients who received RT in addition to chemotherapy were also more likely to have better three-year DSS than those who received chemotherapy alone (28.9% vs. 14.0%, *P*<0.001). Similarly, in the SYSUCC cohort, patients who received chemotherapy plus RT had better three-year OS than chemotherapy alone (62.8% vs. 21.0%, *P*=0.003). The addition of RT to chemotherapy increased median OS among patients with mHNSCC from 10 months to 13 months in the SEER cohort and from 14 months to 29 months in the SYSUCC cohort.

Conclusion: Radiotherapy in addition to chemotherapy significantly improved OS and DSS in patients with mHNSCC.

## Introduction

Head and neck squamous cell carcinoma (HNSCC) is the sixth most common type of cancer worldwide. Globally every year, over 890,000 individuals are diagnosed with HNSCC, with over 450,000 deaths attributed to this cancer 1. Studies have reported that the incidence of distant metastasis (synchronous distant metastasis at first diagnosis or metachronous distant metastasis after curative surgery) in HNSCC varies from 4.2% to 23.8% 2. The 5-year OS rate ranges from 70%-90% for localized disease (stage I or stage II) 3, 4, while that for advanced cases ranges from 10%-40% 5, 6. Patients presenting with distant metastasis are generally considered incurable; these patients undergo palliative treatment to prolong survival and improve quality of life. The prognosis for patients with metastatic HNSCC is dismal, with the median survival ranging from 1 to 12 months 7, 8. These suggest an urgent need to develop novel therapeutic options to improve clinical outcomes in these patients.

Systemic treatment options for patients with mHNSCC are limited. Patients are often treated with palliative chemotherapy consisting of platinum-based doublets or a single-agent cytotoxic drug 9, 10. Although combination chemotherapy enhances response rates compared with single-agent cisplatin or methotrexate, no combination chemotherapy regimen has been demonstrated to improve OS 11. Substantial evidence shows that epidermal growth factor receptor (EGFR) could be used as a therapeutic target in HNSCC 12, 13. The EXTREME trial demonstrated that the addition of cetuximab to platinum/5-FU chemotherapy improved response rates (from 20% to 36%) and prolonged median overall survival (from 7.4 to 10.1 months) for patients with recurrent and metastatic (R/M) disease 14. It is the first study in which any treatment for HNSCC has shown a survival advantage in the R/M setting. The Food and Drug Administration (FDA) and European Medicine Agency (EMA) approved the addition of cetuximab to platinum-based therapy for R/M HNSCC in 2011. Despite widespread EGFR expression in HNSCC tumors, only a small subset of mHNSCC patients experience a survival benefit from cetuximab therapy 15. Recent studies suggest that HNSCC patients may benefit from immune checkpoint inhibitors 16. The optimal treatment strategy for patients with R/M HNSCC is still controversial. The National Comprehensive Cancer Network (NCCN) is of the view that the best management for patients with mHNSCC is enrollment into a clinical trial 10.

Radiation therapy (RT) is a fundamental modality in the treatment of HNSCC. Studies have reported that patients with locally advanced HNSCC can benefit from RT, both for locoregional control and 3-year survival 17. RT is an optimal strategy to use for local control in HNSCC patients who have lost the opportunity for surgical intervention due to the appearance of distant metastasis. However, whether adding RT to systemic therapy will improve the survival of mHNSCC patients remains uncertain. Evidence from studies of other advanced-stage malignant tumors has indicated the potential benefits of RT in local control even when the disease has metastasized. For metastatic urothelial carcinoma of the bladder, high-intensity local treatment of the primary tumor burden increased median OS of 5 months compared with conservative local treatment 18. Daniel R *et al.* demonstrated an improvement in progression-free survival and OS for patients with oligometastatic non-small-cell lung cancer who received local consolidative therapy than those who did not 19. Similarly, Ming-Yuan Chen *et al.* 20 showed in their pilot phase III study that adding RT to systemic treatment in patients with de novo metastatic nasopharyngeal carcinoma resulted in an improvement in two-year OS from 54.5% to 76.4%. Although nasopharyngeal carcinoma is a separate clinicopathological entity, it is also one of the head and neck cancers in broad terms.

Given the overall evidence, we hypothesized that RT could improve survival in patients with distant metastatic HNSCC. Therefore, this study aimed to evaluate the prognosis of patients diagnosed with mHNSCC comparing treatment of chemotherapy plus RT with chemotherapy alone. We used the Surveillance, Epidemiology, and End Results (SEER) database and a Chinese single-institution registry in Sun Yat-sen University Cancer Center (SYSUCC) to evaluate the impact of RT on clinical outcomes for patients with mHNSCC in the era of cetuximab.

## Materials and methods

### Patient Population

This study was approved by the Institutional Review Board (B2022-168-01).

*SEER database Cohort.* For the current analysis, we utilized data from 18 SEER databases of the National Cancer Institute in the United States (US). SEER is a program that collects and reports data on cancer incidence, treatment, and survival from population-based cancer registries, representing approximately 28% of the US population. The 18 registries in SEER-18 include approximately 25% of the white population, 26% of the black population, 38% of the Hispanic population, 44% of the American Indians and Alaska (A/ PI) population, 50% of Asians and 67% of Hawaiian/ Pacific Islanders. The 18 SEER registries comprising Atlanta, Detroit, Greater California, Greater Georgia, Hawaii, Iowa, Kentucky, Los Angeles, New Mexico, New Jersey, Rural Georgia, states of Connecticut, San Francisco-Oakland, Seattle-Puget Sound, San Jose-Monterey, the Alaska Native Tumor Registry, Louisiana and Utah were used for this analysis. This was a retrospective analysis of patients who presented with distant metastatic disease between 2010 and 2015 and were included in the SEER registries. All patients were staged based on the 7^th^ edition of the American Joint Committee on Cancer staging system (2010-2015).

*Sun Yat-sen University Cancer Center Cohort.* The Sun Yat-sen University Cancer Center (SYSUCC) is among the first batch of cancer multidisciplinary treatment pilot hospitals set up by the National Health Commission in China. To ensure our results are generalizable and representative, we collected similar cases as above from SYSUCC. Most patients in the SYSUCC cohort had metachronous distant metastasis after curative surgery. These cases were used to make independent comparisons with respect to the Asian population, given the limited number of Asians in the SEER database.

### Patients Selection

**Figure 1:**
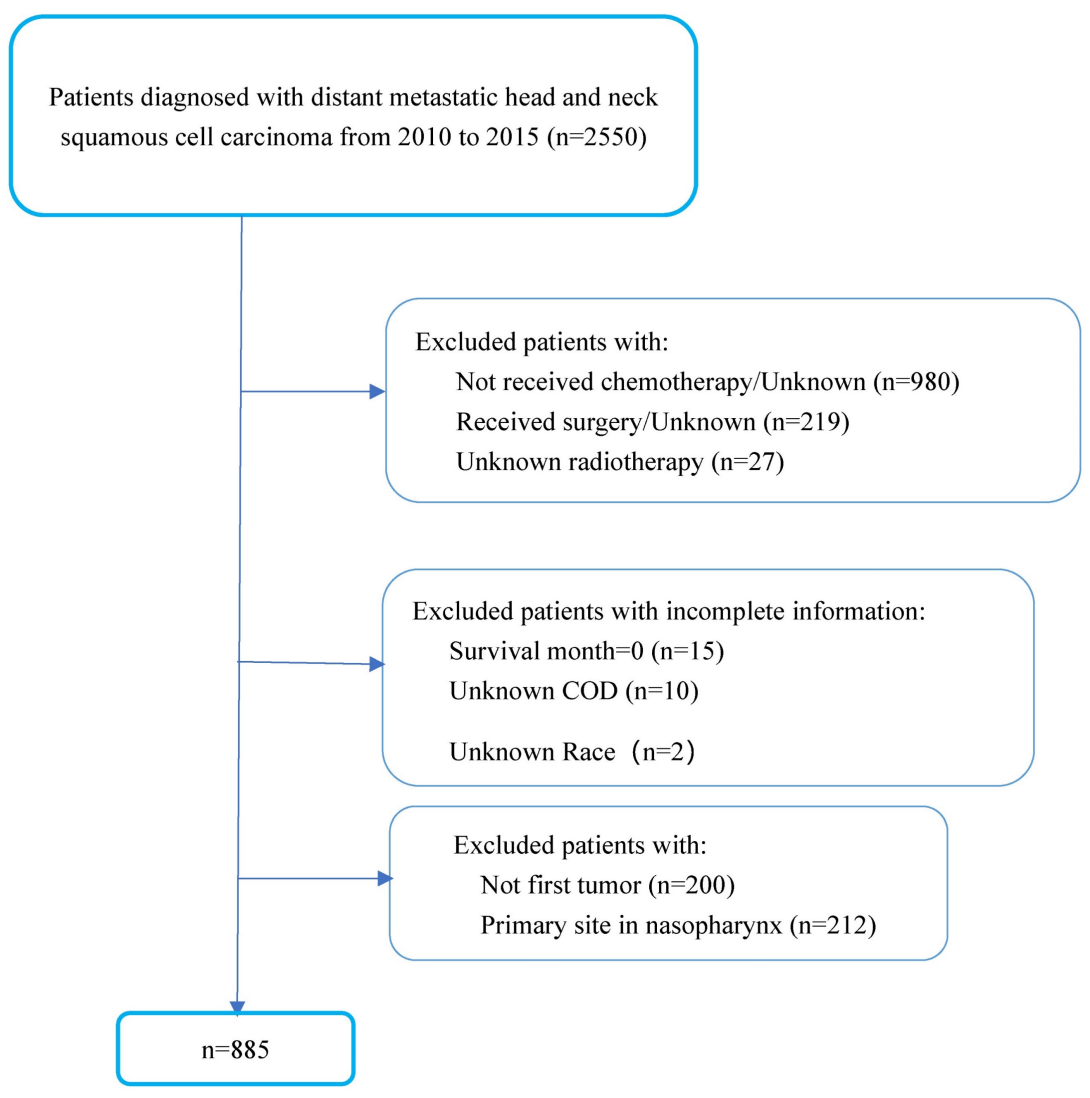
Patients' selection flowchart.

We included 885 patients diagnosed with mHNSCC between 2010 and 2015 from the SEER databases of the National Cancer Institute in the US and 60 patients diagnosed with mHNSCC between 2010 and 2020 from SYSUCC. The period of the study reflected the use of cetuximab in the treatment of HNSCC. In the SEER cohort, we excluded patients who did not receive chemotherapy or had no known records of receiving RT. To better represent the poor health status of patients with advanced-stage disease, patients who had been treated with surgery were excluded from the study. Cases of mHNSCC with no first tumor or primary site in the nasopharynx were excluded since these were not the cases of interest in this study. Patients with incomplete information were also excluded (Figure 1). In the SYSUCC cohort, recruited criteria were consistent with the SEER cohort except that patients who had undergone curative surgery were also included due to the limited number of cases.

Malignant cases were identified by the International Classification of Diseases (ICD-O-3) to classify tumor histology and tomography. We included all patient's diagnoses with mHNSCC reported to the SEER program between 2010 and 2015, as defined by ICD-O-3 morphology codes: 8052, 8070, 8071, 8072, 8073, 8074, 8076, and 8083. Anatomic locations of cases were divided into four sites (oral cavity, oropharynx, hypopharynx and larynx). In this study, oral cavity was defined by ICD-O-3 site recodes C00.1, C01.9, C02.0, C02.1, C02.2, C02.3, C02.4, C02.8, C02.9, C03.0, C03.1, C03.9, C04.0, C04.9, C05,0, C05.1, C05.2, C06.0, C06.1, C06.2, C07.9, C08.0, C08.9, C09.0, C09.1, C09.8 and C09.9; oropharynx included C10.0, C10.1, C10.2, C10.3, C10.8 and C10.9; hypopharynx by ICD-O-3 codes C13.0, C13.1, C13.2. C13.8, and C13.9; and the larynx by ICD-O-3 codes C32.0, C32.1, C32.2, C32.8 and C32.9.

### Statistical Analysis

All analyses were conducted using Statistical Package for the Social Sciences (SPSS) version 25.0 software (IBM Corporation, Armonk, NY) and figures were plotted in the GraphPad PRISM software (GraphPad, San Diego, CA, USA). Descriptive statistics were used to summarize the following baseline characteristics of mHNSCC cases: age of diagnosis, gender, race, primary tumor site, initial tumor-node-metastasis (TNM) staging classification, treatment method, insurance status, and marital status.

A bivariate comparison of RT plus chemotherapy vs. chemotherapy alone for continuous and categorical variables was performed using Welch's t-test, Wilcoxon-Mann-Whitney test, or Pearson's chi-squared test where appropriate. Univariate and multivariable logistic regression was used to identify factors associated with the use of RT plus chemotherapy vs. chemotherapy alone. Kaplan-Meier analysis was used to estimate survival probabilities and the log-rank test was used to compare Kaplan-Meier curves. Time-to-event was defined as the time from mHNSCC diagnosis to the last follow-up or death. A *P* value of 0.05 or less was considered statistically significant. All *P* values were accurate to three decimal places.

### Vital Status

OS and DSS were obtained as of December 31, 2015, from the SEER database using active and passive surveillance of national and statewide databases. All patients in the SYSUCC cohort were diagnosed from January 1, 2010, through December 31, 2020. Survival status diagnosis and treatment information are obtained from the case registration and follow-up systems of the SYSUCC. OS was defined as the time from the date of diagnosis to the date of death due to any reason. DSS refers to the time from diagnosis to death due to the disease.

## Results

### Patients Status and Application of Radiotherapy

A total of 885 patients in the US SEER cohort and 60 patients in the Chinese SYSUCC cohort who met the eligibility criteria were included in the final analysis. The baseline characteristics of each cohort are reported in Table 1 and Table 2, respectively. A total of 689 deaths were recorded (22.1% censored) in the SEER cohort and 35 deaths were recorded (9 cases censored, 17 cases still alive) in the SYSUCC cohort. For the SEER cohort, 600 patients received RT plus chemotherapy and 285 received chemotherapy alone. Radiotherapy was used in 67.8% of patients and its application remained relatively constant (64.9-73.4%) over time (Figure S1). The use of RT in the SYSUCC cohort was consistent with that of the SEER cohort, a total of 40 patients (66.7%) received RT.

### Patients Characteristics

Baseline characteristics of the SEER cohort are shown in Table 1. The data showed that mHNSCC frequently occurred in those aged 50 years and older, white non-Hispanic male patients. Stratification by primary subsites demonstrated that the occurrence of mHNSCC in the oral cavity, oropharynx, hypopharynx, and larynx was 59.7%, 7.9%, 6.3%, and 26.1%, respectively. At initial diagnosis, most patients were T2 and N2 stage, or more advanced. The majority of patients were insured; however, less than half of them were in common-law marriages. The median life expectancy for the whole cohort after diagnosis of mHNSCC was 12 months. There were no statistically significant differences between the two treatment groups in terms of gender, age, race, primary site, TNM classification, marital status, and insurance status. The baseline data of the SYSUCC cohort was slightly different from that of the SEER cohort (Table 2). The majority of patients were male (96.7%). Stratification by primary subsites showed that mHNSCC in Chinese patients was mostly found in the hypopharynx (48.3%). The prevalence of the primary tumor site in the oral cavity, oropharynx, and larynx was 15.0%, 10.0%, and 26.7%, respectively. Similarly, most patients at initial diagnosis were advanced T stage (T3~T4, 53.3%) and N stage (N2~N3, 66.7%). The median survival time of this cohort was 23 months.

### Survival Analysis

**Figure 2:**
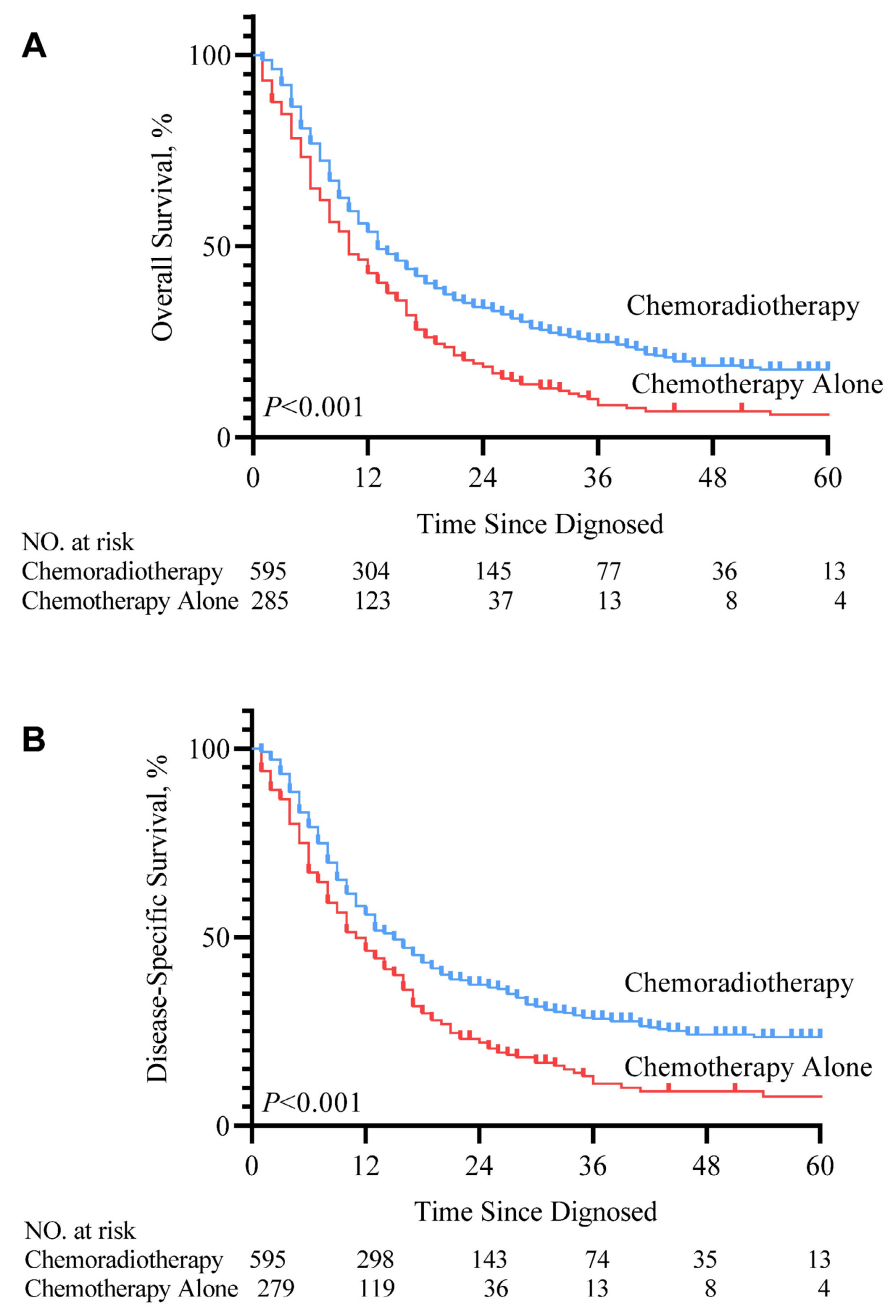
KM overall survival curve (A) and disease-specific survival curve (B) according to the treatment type cohort 1.

**Figure 3:**
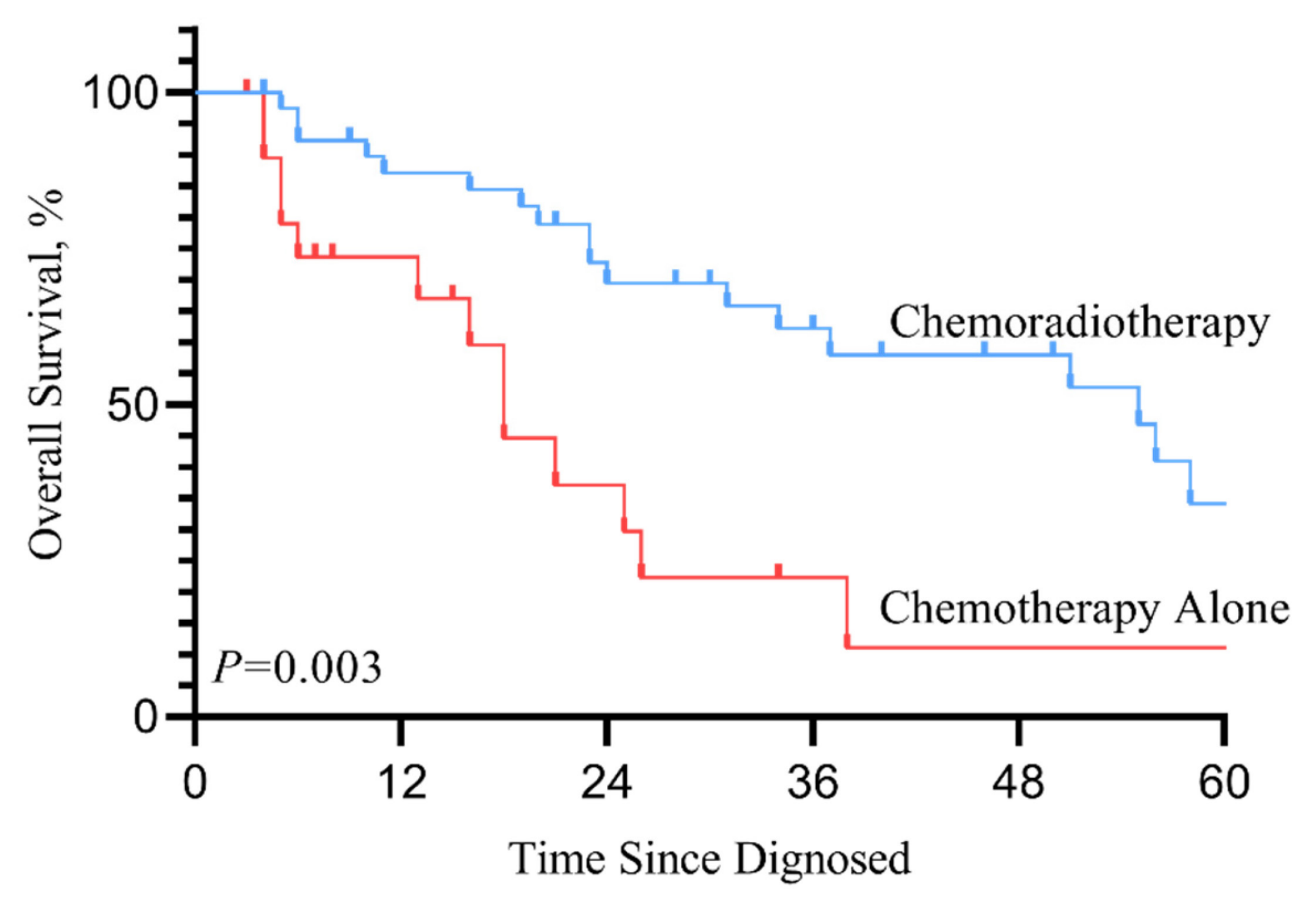
Kaplan-Meier overall survival curve according to treatment type in Chinese cohort 2.

In the SEER cohort, patients who received RT in addition to chemotherapy after diagnosis with mHNSCC had significant improvement in life expectancy compared to those who received chemotherapy alone (median survival months, 10 vs. 13 months). Kaplan-Meier survival curve (Figure 2. *P*<0.001) showed that for patients with mHNSCC, RT plus chemotherapy was associated with significant improvement in OS and DSS compared to chemotherapy alone (three-year OS, 25.1% vs. 10.4%, *P*<0.001; three-year DSS, 28.9% vs. 14.0%, *P*<0.001) (Table 3). Similarly, of the 60 patients diagnosed with distant metastasis HNSCC from 2010 to 2020 at SYSUCC, those who received RT in addition to chemotherapy showed prolonged survival. Kaplan-Meier survival curves for the treatment groups are shown in Figure 3. Patients who received RT plus chemotherapy had better life expectancy than those who received chemotherapy alone (three-year OS, 62.8% vs. 21.0%, *P*=0.003). The detailed survival outcomes of SYSUCC patients are shown in Table 4. We did not evaluate the differences among primary tumor sites in the SYSUCC cohort due to a limited number of cases for subgroup analysis.

**Figure 4:**
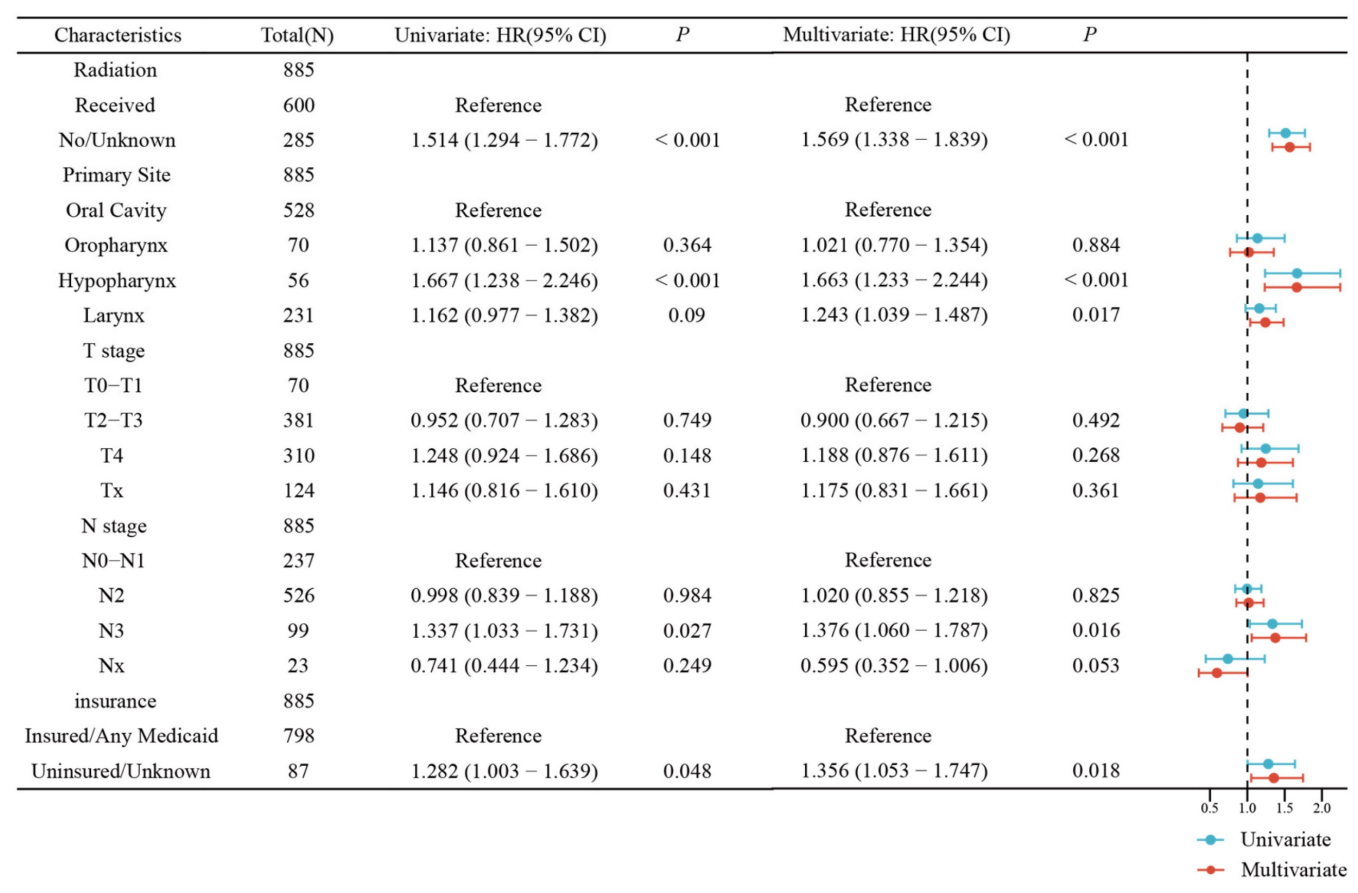
Univariate and multivariate Cox regression analysis forest plot of the head and neck (mHNSCC) patients stratified by tumor sites from cohort 1.

**Figure 5:**
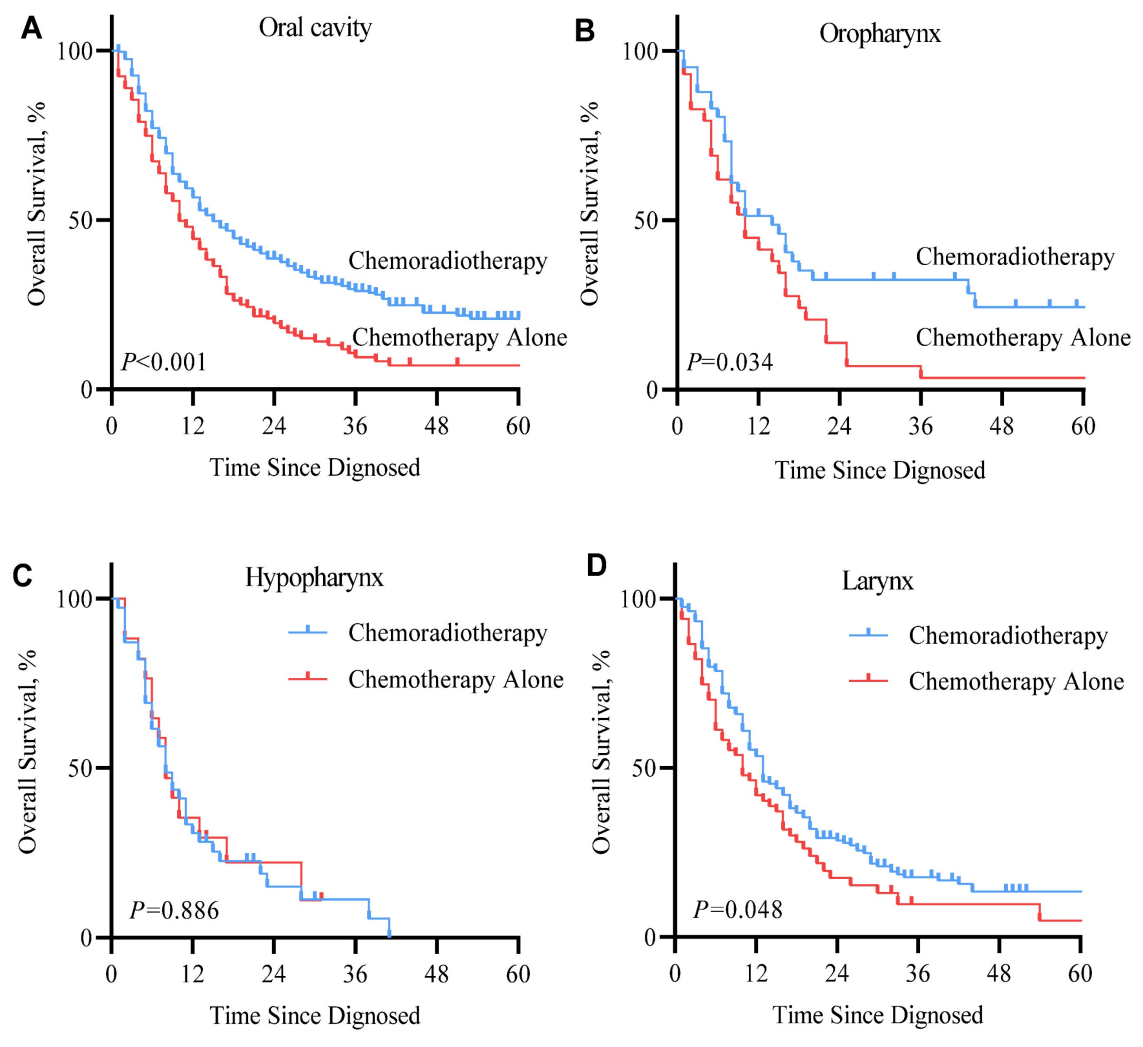
Kaplan-Meier overall survival curve of metastatic squamous cell carcinoma of the head and neck (mHNSCC) patients stratified by tumor sites from cohort 1.

The univariable analysis showed that RT was associated with better OS. In the univariate and multivariate Cox regression analysis forest plot of the SEER cohort (Figure 4), RT was significantly and independently associated with better OS compared with chemotherapy alone (Hazard ratio, HR 1.57, 95% confidence interval, CI 1.34-1.84, *P* <0.001), which was similar to that of the univariable analysis. The KM overall survival analysis demonstrated that patients presenting with less tumor invasion (T0~T1 and N0~N1) had better OS (Figure S2). Insured patients had better life expectancy. It should be noted that the primary subsite had a significant impact on the prognosis of patients in all three analyses. In the multivariable Cox regression model, mHNSCC in the hypopharynx had the worst prognosis than the oral cavity (oral cavity versus hypopharynx: HR=1.65, 95% CI 1.22-2.23, *P=*0.001), followed by the larynx. There was no significant difference comparing the oral cavity and the oropharynx. However, patients with primary tumors in the oral cavity, oropharynx, and larynx had a better prognosis in the RT group, as shown in the Kaplan-Meier survival curves (Figure 5). Gender, age, race, and marital status were not significantly associated with patients' outcomes in either univariable or multivariable analyses. In the SYSUCC cohort, the combination of RT plus chemotherapy was significantly and independently associated with better OS compared to chemotherapy alone (HR 3.14, 95% CI 1.34~7.37, *P* =0.008) (Figure S3). However, T stage, N stage, and primary cancer sites were not independent risk factors for survival in patients with SYSUCC in the multivariable Cox regression model (Table S1).

## Discussion

The combination of systemic therapy and supportive care remains the mainstay of treatment for patients with mHNSCC. Radiation therapy has been demonstrated to provide good local control and improved survival in some subtypes of cancer with distant metastasis 19-21. The effect of adding RT to systemic therapy for the treatment of mHNSCC was evaluated in this study. Utilizing SEER and SYSUCC databases, we demonstrated that adding RT to systemic chemotherapy significantly improved survival in patients with mHNSCC compared to systemic chemotherapy alone: median OS increased from 10 months to 13 months and 3-year OS improved from 10.4% to 25.1% in SEER cohort; for the SYSUCC cohort, median OS increased from 17 months to 35 months and 3-year OS improved from 21.0% to 62.8%.

Several previous studies have evaluated the role of RT in the management of metastatic HNSCC. A National Cancer data-based study demonstrated that the combination of locoregional control (included surgical resection of the primary, external beam radiation alone, or external beam radiation with isotopes or implants) with systemic therapy was superior to systemic therapy alone for survival in patients with distant metastatic head and neck cancer (HR =0.73, *P*<0.001) 22. Another SEER-based study 23 observed that in patients with mHNSCC, mean survival was 8.4, 18, 31, and 40 months with no local treatment, RT alone, surgery alone, and surgery with RT, respectively. Though that study did not involve systemic therapy, the results still emphasized the importance of local control in patients with mHNSCC. Based on a retrospective single-institution analysis, Ampil *et al.*^24^ observed that irradiation showed superiority compared with systemic therapy alone for synchronous M1 head and neck cancer (median survival month: 14 months vs. 5.5 months; 60-day mortality rate 0% vs. 50%). Zachary *et al.* 25 reported that most mHNSCC patients benefitted from high-intensity RT (defined as radiation doses ≥ 60Gy) for the primary site of the tumor. In clinical practice, hyper-fractionated palliative radiation is beneficial for mHNSCC patients with poor status, who are not suitable for high-dose RT 25, 26. Our study findings showed that mHNSCC patients experienced prolonged median survival, as well as apparent benefit in both 3-year OS and DSS rates derived from RT in addition to systemic treatment.

Compared to previous studies, our study design has several advantages. To our knowledge, this is the first SEER-based prognostic study of the effectiveness of RT in mHNSCC patients who have undergone systemic therapy. This is also the first and largest SEER population-based study of these patients following FDA approval of the use of cetuximab. Furthermore, this is the first study to analyze and compare the differences in the prognosis of mHNSCC patients in the U.S. and China. The diverse nature of these cohorts strengthens the results and broadens the generalizability of the findings.

The beneficial treatment effect of RT in addition to systemic therapy in mHNSCC patients may be attributed to several factors. The most likely and simplest explanation is that RT can often achieve ideal local control even when the disease has distant metastasis 18, 19, 27. Considering the nature of the disease's anatomy and its treatment, HNSCC can affect function in vital areas that facilitate breathing, eating, and speaking, in addition to blood vessels and nerves necessary for survival. Local control for mHNSCC patients reduces mortality from locoregional progression. Besides, activation of natural anti-tumor responses may also contribute to the beneficial effects of RT. Available evidence supports the concept that radiation-induced cell death could promote dendritic cell uptake and produce tumor-derived antigens 28, 29. RT can also enhance anti-tumor immune response by promoting cytotoxic T lymphocytes, professional antigen-presenting cells, and macrophage-releasing cytokines and tumor antigens to the irradiated micro-environment 30. In addition, original tumor cells may recapture aggressive segments by self-seeding from circulating tumor cells, resulting in a further enhancement of tumor growth through the action of seed-derived signals and tumor-produced growth factors and cytokines 31, 32. RT can interrupt steps in the self-seeding process, decrease cell-to-cell heterogeneity, and reduce the overall subclone burden 33. Thus, RT benefits mHNSCC patients as an adjunct to chemotherapy.

The benefit of RT was observed across nearly all subsite groups. In the SEER cohort, patients with primary oral cavity tumors benefited most obviously from RT, median survival was 13.5 months vs. 10 months for patients who received combined RT with systemic therapy vs. systemic therapy alone (*P*<0.001). Tumors in the oral cavity can affect function in vital areas that facilitate the ability to eat, drink, chew, and swallow 34, which may have a direct impact on physiology and psychology. Harsh *et al.* indicated that the addition of RT resulted in substantially better survival in locally advanced oral cancer 35. Therefore, oral cavity HNSCC patients with well-controlled local disease will have a significantly better survival rate. In patients with mHNSCC whose tumor originated from the oropharynx and larynx, the addition of RT resulted in moderate improvement in survival (2- and 3-month improvement from 10 months, respectively). However, hypopharynx cases had the worst prognosis than other sites (median survival of 8 months), and the addition of RT to systemic therapy was not significantly associated with a different prognosis. Previous studies have reported that compared with HNSCC primary in the oral cavity, the primary site at the hypopharynx was strongly associated with distant metastasis (hypopharynx vs. oral cavity, OR=2.27) 36, 37. For locally advanced HNSCC patients who following treatment failure, patients with hypopharynx HNSCC have the highest cumulative incidence of distant metastasis and poorest median survival compared to other sites ( 30.1% DM, while the other sites all less than 20%; 5-year OS, 48.9% while the other sites all higher than 50%) portends hypopharynx HNSCC has a greater aggressive nature 37, which may partly explain hypopharynx mHNSCC patients' inferior prognosis and response to RT. Based on the SYSUCC cohort, the majority of cases were metastatic after curative surgery, with cases originating in the hypopharynx accounting for the majority (*N*=29, 48.3%). This also explains the dismal outcome of mHNSCC patients with tumors that originated from the hypopharyngeal region.

Notably, there are some differences in terms of primary tumor site and long-term survival between our two cohorts. In the SEER cohort, the majority of cases originated from the oral cavity, whereas in the SYSUCC cohort, hypopharynx HNSCC accounted for the largest proportion. Higher HPV infection rates of HNSCC patients in the U.S. (U.S. 65.4% vs. China 24.7%) may have contributed to more oral cavity cases in the SEER cohort 38, 39. Besides, patients enrolled in the SEER cohort were all diagnosed with de novo distant metastatic HNSCC, while the majority of SYSUCC patients were metachronous distant metastasis after curative surgery. Hypopharyngeal tumors accounted for the majority of patients in the SYSUCC cohort, which is consistent with the above concept that hypopharynx carcinoma has a more aggressive nature with a high rate of systemic metastases. In terms of long-term survival, the preponderance of patients with a history of surgery may account for the better long-term prognosis of SYSUCC patients. Nevertheless, our overall results suggest that RT has immense benefits for patients with mHNSCC.

Several limitations need to be considered. The major limitation of our study is its retrospective nonrandomized cohort design. As with any population-based retrospective study, patients were not enrolled and evaluated prospectively within the constraints of a controlled clinical trial, thus there is an inherent patient selection bias that must be acknowledged for this study. The issue of immortal time bias (also known as survivor treatment selection bias) is difficult to overcome when comparing patients who received RT with those who did not, because the patients who were to receive RT had to survive long enough to complete treatment. Furthermore, the SEER database does not record information regarding RT details (fields, dose, fractionation, distant lesion/ locoregional control), HPV status, and extent of metastatic disease data. As a result of this and the relatively small sample size of the SYSUCC cohort, we could not confirm the impact of radiotherapy plans, HPV infection, or the extent of metastatic disease in our study 40.

Despite these limitations, SEER registries constitute a representative sample of the US mHNSCC patient population 41 and with the addition of data from the SYSUCC cohort, this study provides very valuable data for improving the prognosis of mHNSCC patients.

## Conclusion

In this retrospective study, we found that mHNSCC patients receiving combined RT and chemotherapy had prolonged median survival as well as better OS and DSS than chemotherapy alone. Despite the limitations of this observational data, our study provides comprehensive information for further definitive prospective clinical trials to evaluate the optimal timing and dose of RT in mHNSCC patients. In the absence of contraindications, we suggest that all HNSCC patients with distant metastatic disease probably should receive RT in addition to systemic chemotherapy.

## Supplementary Material

Supplementary figures and table.

## Figures and Tables

**Table 1:** Characteristics of metastatic squamous cell carcinoma of the head and neck (mHNSCC) patients in the SEER cohort from 2010 to 2015.

Patient Characteristics	No. (%)			
	Whole cohort (n=885)	Chemotherapy plus RT (n=600)	Chemotherapy Alone (n=285)	P value
Gender				0.828
Male	723 (81.7)	489 (81.5)	234 (82.1)	
Female	162 (18.3)	111 (18.5)	51 (17.9)	
Age, y				0.320
Median (IQR)	61.0 (55.0~68.0)	61.0 (55.0~67.0)	63.0 (56.0~69.0)	
Mean (95%CI)	61.8 (61.2~62.4) (61.2~62.4)	61.5 (30.7~62.2)	62.5 (61.3~63.6)	
<50	84 (9.5)	58 (9.7)	26 (9.1)	
50-59	289 (32.7)	204 (34.0)	85 (29.8)	
60-69	334 (37.7)	227 (37.8)	107 (37.5)	
>69	178 (20.1)	111 (18.5)	67 (23.5)	
Race				0.170
White	689 (77.9)	457 (76.2)	232 (81.4)	
Black	160 (18.1)	115 (19.2)	45 (15.8)	
Other	36 (4.1)	28 (4.7)	8 (2.8)	
Primary Site				0.269
Oral Cavity	528 (59.7)	356 (59.3)	172 (60.4)	
Oropharynx	70 (7.9)	41 (6.8)	29 (10.2)	
Hypopharynx	56 (6.3)	39 (6.5)	17 (6.0)	
Larynx	231 (26.1)	164 (27.3)	67 (23.5)	
T classification				0.353
T0~T1	70 (7.9)	50 (8.3)	20 (7.0)	
T2~T3	381 (43.1)	268 (44.7)	113 (39.6)	
T4	310 (35.0)	203 (33.8)	107 (37.5)	
Tx	124 (14.0)	79 (13.2)	45 (15.8)	
N classification				0.088
N0~N1	237 (26.8)	162 (27.0)	75 (26.3)	
N2	526 (59.4)	348 (58.0)	178 (62.5)	
N3	99 (11.2)	77 (12.8)	22 (7.7)	
Nx	23 (2.6)	13 (2.2)	10 (3.5)	
Spanish-Hispanic-Latino				0.500
NO	789 (89.2)	532 (88.7)	257 (90.2)	
Yes	96 (10.8)	68 (11.3)	28 (9.8)	
Marriage status				0.392
Married	373 (42.1)	247 (41.2)	126 (44.2)	
Other	512 (57.9)	353 (58.8)	159 (55.8)	
Insurance status				0.806
Insured	798 (90.2)	540 (90.0)	258 (90.5)	
Uninsured/Unknown	87 (9.8)	60 (10.0)	27 (9.5)	

**Table 2:** Characteristics of metastatic squamous cell carcinoma of the head and neck (mHNSCC) patients in cohort 2.

Patient Characteristics	No. (%)			
	Whole cohort (n=60)	Chemotherapy plus RT (n=40)	Chemotherapy Alone (n=20)	P
Gender				0.548
Male	58 (96.7)	38 (95.0)	20 (100.0)	
Female	2 (3.3)	2 (5.0)	0 (0.0)	
Age, y				0.616
Median (IQR)	55.8 (50.3~62.0)	55.4 (49.0~61.8)	56.6 (53.0~62.0)	
<59	39 (65.0)	26 (65.0)	13 (65.0)	
≥60	21 (35.0)	14 (35.0)	7 (35.0)	
Primary Site				0.797
Oral Cavity	9 (15.0)	5 (12.5)	4 (20.0)	
Oropharynx	6 (10.0)	4 (10.0)	2 (10.0)	
Hypopharynx	29 (48.3)	19 (47.5)	10 (50.0)	
Larynx	16 (26.7)	12 (30.0)	4 (20.0)	
T classification				0.361
T1~T2	19 (31.7)	15 (37.5)	4 (20.0)	
T3~T4	32 (53.3)	20 (50.0)	12 (60.0)	
Tx	9 (15.0)	5 (12.5)	4 (20.0)	
N classification				0.367
N0~N1	11 (18.3)	9 (22.5)	2 (10.0)	
N2~N3	40 (66.7)	25 (52.5)	15 (75.0)	
Nx	9 (15.0)	10 (25.0)	3 (15.0)	

**Table 3:** Cumulative overall survival and disease-specific survival in metastatic squamous cell carcinoma of the head and neck (mHNSCC) patients from cohort 1.

Survival	Chemotherapy plus radiotherapy	Chemotherapy Alone	P
Total cases, N	600	285	
Dead cases, N (%)	443 (73.8)	246 (86.3)	<0.001
Median Survival (months)	13	10	
One-year OS	56.0%	46.6%	0.009
(month, 95%CI)	(52.0%~60.0%)	(40.8%~52.4%)	
Three-year OS	25.1%	10.4%	<0.001
(month, 95%CI)	(21.4%~28.9%)	(6.4%~14.5%)	
Five-year OS	17.8%	6.2%	<0.001
(month, 95%CI)	(14.0%~21.6%)	(2.4%~10.0%)	
One-year DSS	58.6%	50.0%	0.017
(month, 95%CI)	(54.6%~62.6%)	(44.1%~55.9%)	
Three-year DSS	28.9%	14.0%	<0.001
(month, 95%CI)	(24.8%~32.8%)	(9.0%~18.9%)	
Five-year DSS	23.8%	8.2%	<0.001
(month, 95%CI)	(19.5%~28.1%)	(3.9%~12.5%)	

**Table 4:** Cumulative overall survival in metastatic squamous cell carcinoma of the head and neck (mHNSCC) patients from cohort 2.

Survival	Chemotherapy plus radiotherapy	Chemotherapy Alone	P
Total cases, N	40	20	
Dead cases, N(%)	22 (55.0)	13 (65.0)	0.459
Median Survival (months)	35.2	17.2	
One-year OS	87.2%	72.2%	0.206
(month, 95%CI)	(77.0%~98.3%)	(56.3%~96.4%)	
Three-year OS	62.8%	21.0%	0.003
(month, 95%CI)	(47.5%~81.3%)	(8.4%~59.5%)	
Five-year OS	36.3%	10.5%	0.064
(month, 95%CI)	(17.5%~62.7%)	(2.0%~61.0%)	

## Abbreviations

mHNSCC: metastatic squamous cell carcinoma of the head and neck
RT: radiotherapy
SEER: The Surveillance, Epidemiology, and End Results
SYSUCC: Sun Yat-sen University Cancer Center
OS: overall survival
DSS: disease-specific survival
HNSCC: head and neck squamous cell carcinoma
EGFR: epidermal growth factor receptor
R/M: recurrent and metastatic
FDA: Food and Drug Administration
EMA: European Medicine Agency
NCCN: National Comprehensive Cancer Network
ICD-O-3: The 3rd edition of International Classification of Diseases for Oncology
TNM: tumor-node-metastasis
HR: hazard ratio
