# Supplementary material for: Radiation Therapy Improves Survival in Patients with Distant Metastatic Head and Neck Squamous Cell Carcinoma: A Retrospective Study
Source: J Cancer. 2025 Jan 1;16(3):996–1007. doi: 10.7150/jca.105254 (PMC11705048; doi:10.7150/jca.105254)
Supplement: Supplementary file 1 — Supplementary figures and table. [file jcav16p0996s1.pdf]

Supplement information

Figure S1. Constant proportion of RT use in SEER database mHNSCC patient by year.

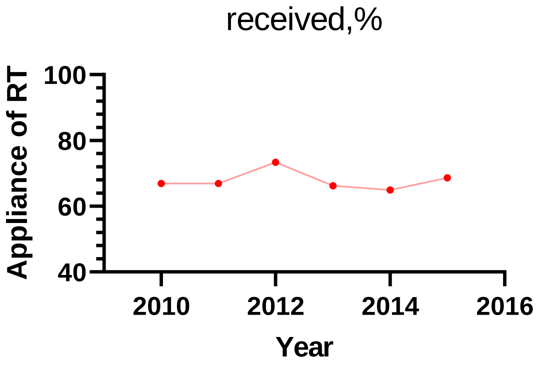

Figure S2. KM overall survival curves of mHNSCC patients stratified by different clinical factors from cohort 1.

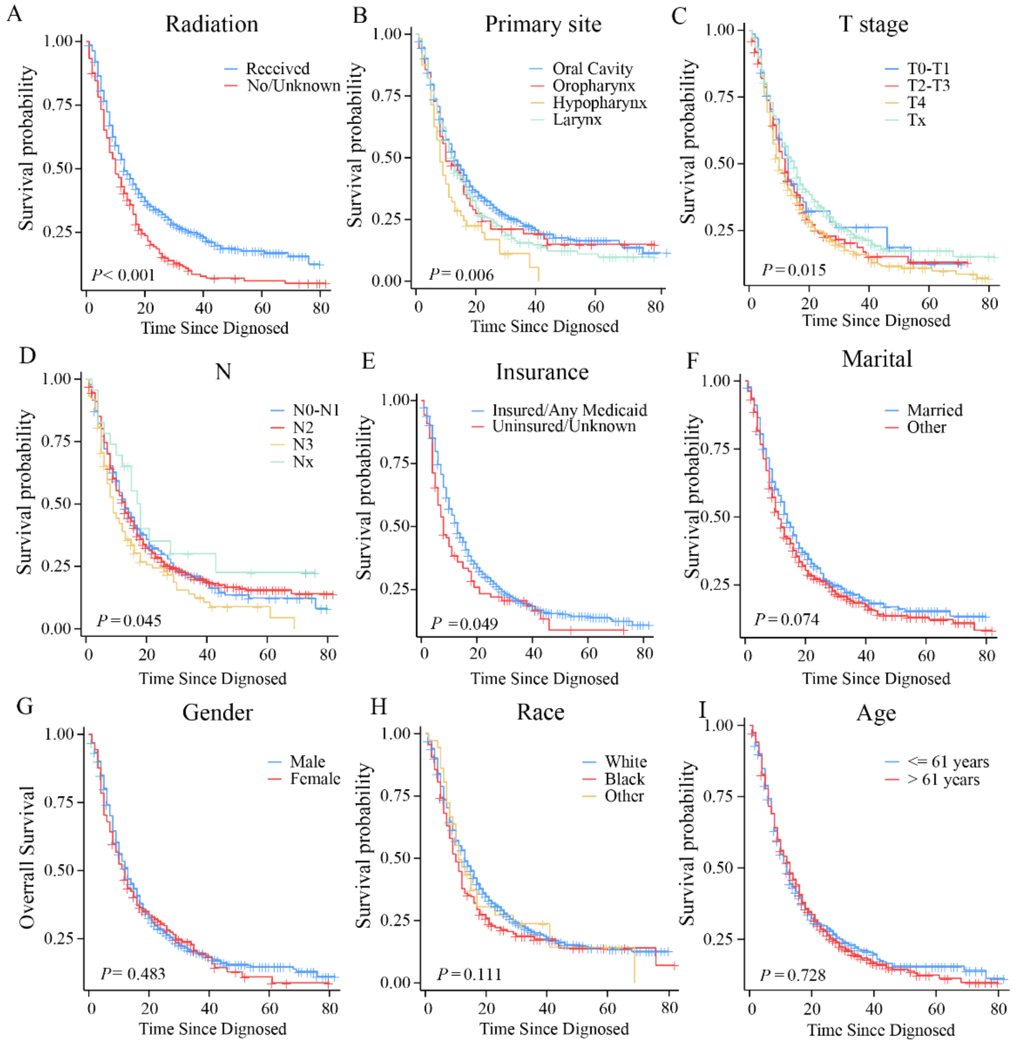

Figure S3. KM overall survival curves of mHNSCC patients stratified by tumor sites from cohort2.

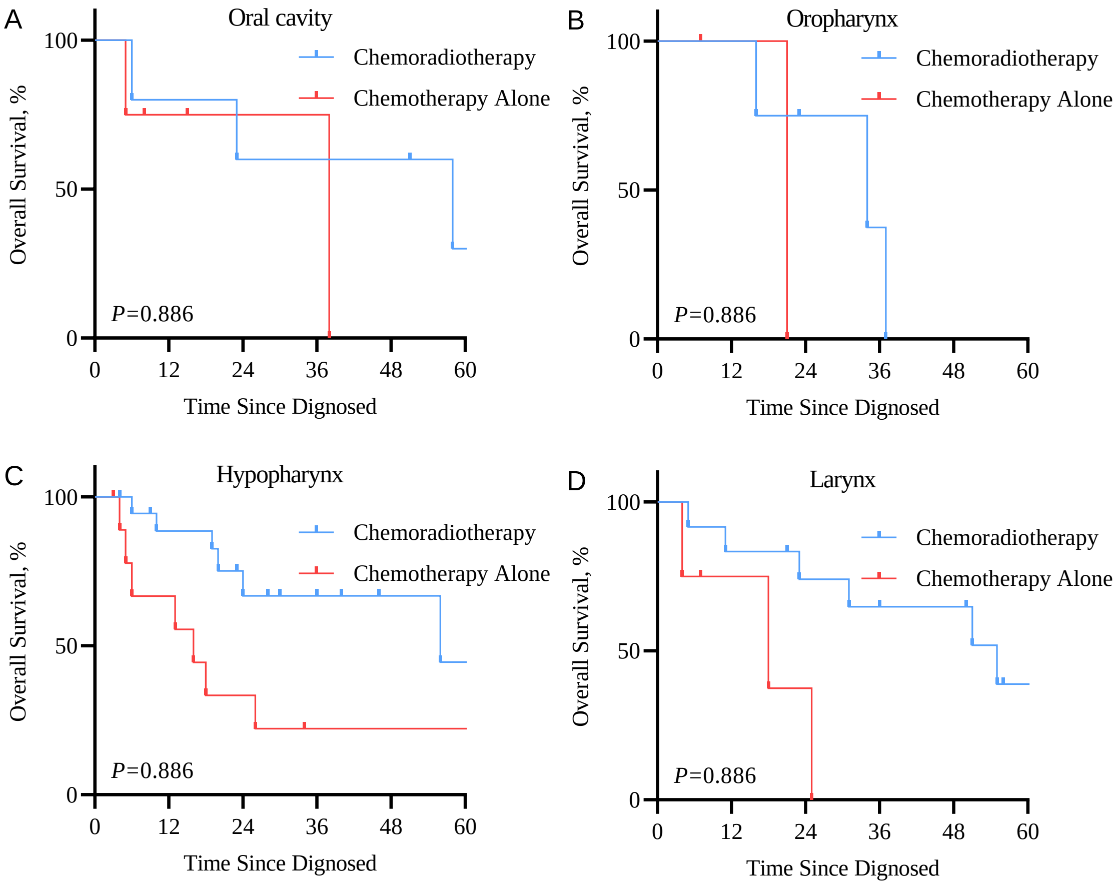

Table S1. Cox regression analysis of prognostic factors in metastatic squamous cell carcinoma of the head and neck (mHNSCC) patients from the cohort 2.

	KM Log-rank Test	Multivariate cox PH		
	P	HR	95%CI	*P*
Radiotherapy				
Received			Reference	
Not received	0.003	3.14	1.34~7.37	0.008
N Stage				
N0~N1	0.461		Reference	
N2~N3	0.214	2.22	0.71-6.92	0.170
Nx	0.349	1.50	0.18-12.44	0.709
T Stage				
T1~T2	0.757		Reference	
T3~T4	0.831	0.85	0.33-2.91	0.734
Tx	0.831	1.50	0.18-12.44	0.709

Primary Site				
Oral Cavity	0.822		Reference	
Oropharynx	0.954	1.49	0.39-5.67	0.559
Hypopharynx	0.352	0.87	0.29-2.56	0.797
Larynx	0.644	1.23	0.39-3.89	0.846
